# Single Molecule Fluorescence Image Patterns Linked to Dipole Orientation and Axial Position: Application to Myosin Cross-Bridges in Muscle Fibers

**DOI:** 10.1371/journal.pone.0016772

**Published:** 2011-02-08

**Authors:** Thomas P. Burghardt

**Affiliations:** Department of Biochemistry and Molecular Biology and Department of Physiology and Biomedical Engineering, Mayo Clinic Rochester, Rochester, Minnesota, United States of America; University of Maribor, Slovenia

## Abstract

**Background:**

Photoactivatable fluorescent probes developed specifically for single molecule detection extend advantages of single molecule imaging to high probe density regions of cells and tissues. They perform in the native biomolecule environment and have been used to detect both probe position and orientation.

**Methods and Findings:**

Fluorescence emission from a single photoactivated probe captured in an oil immersion, high numerical aperture objective, produces a spatial pattern on the detector that is a linear combination of 6 independent and distinct spatial basis patterns with weighting coefficients specifying emission dipole orientation. Basis patterns are tabulated for single photoactivated probes labeling myosin cross-bridges in a permeabilized muscle fiber undergoing total internal reflection illumination. Emitter proximity to the glass/aqueous interface at the coverslip implies the dipole near-field and dipole power normalization are significant affecters of the basis patterns. Other characteristics of the basis patterns are contributed by field polarization rotation with transmission through the microscope optics and refraction by the filter set. Pattern recognition utilized the generalized linear model, maximum likelihood fitting, for Poisson distributed uncertainties. This fitting method is more appropriate for treating low signal level photon counting data than χ^2^ minimization.

**Conclusions:**

Results indicate that emission dipole orientation is measurable from the intensity image except for the ambiguity under dipole inversion. The advantage over an alternative method comparing two measured polarized emission intensities using an analyzing polarizer is that information in the intensity spatial distribution provides more constraints on fitted parameters and a single image provides all the information needed. Axial distance dependence in the emission pattern is also exploited to measure relative probe position near focus. Single molecule images from axial scanning fitted simultaneously boost orientation and axial resolution in simulation.

## Introduction

Single molecule detection characterizes individual states of a system providing the “bottom-up” description that can be uniquely formulated and tested without the ambiguities inherent in ensemble derived observations [1]. The approach has also lead to surprising new insights in optical imaging such as point object localization at resolution below diffraction limit [2], [3] and the direct detection of the characteristic polarized dipolar emission [4]. The latter links dipole orientation to a spatially resolved emission pattern. We wish to exploit this property to accomplish single molecule orientation detection from an in-focus image with the goal to extract maximal information content from the minimum number of collected photons. The spatial distribution of the emitted light given by the point spread function (PSF) will be mined for its dipole orientation information. The approach is distinct from a traditional one where orthogonal polarized intensities from the single emitter are separated by an analyzer then compared in a ratio of intensities. The PSF mining approach simplifies emission side microscope hardware by requiring just the high spatial resolution CCD camera hence it enables efficient use of collected photons, however, more effort is expended on the analysis of each single molecule image.

Light is emitted by a probe in the aqueous medium but near the glass/aqueous interface formed by the coverslip of a high numerical aperture (NA) oil immersion microscope objective. The interface substantially affects the emitted light it transmits [5] before collection by the objective. Plane waves representing the collected light are refracted into parallel propagating waves that conserve their electric field polarization relative to the meridional plane upon passage through the objective. The meridional plane contains the incident and refracted plane waves and the optical axis. Parallel light transmits a dichroic filter set then is converged into an image on the CCD camera by the tube lens. The tube lens is a low aperture lens that with refraction likewise conserves the electric field polarization relative to the meridional plane. The polarization conserving refractions imply information encoded in emission polarization can be decoded at the detector recognizing that correction is needed for the high NA objective [6]. This is the basis for the traditional analyzer separated orthogonal polarized intensities from a single emitter that are compared in a polarization ratio. Alternatively, polarized emission from the dipole converts to a spatial representation at the objective back focal plane [7], [8] that the tube lens images as the PSF at the camera. We devolve the PSF into 6 basis patterns that, in linear combination, specify any single molecule emission pattern. Given the basis patterns, we invert an observed image to deduce the basis pattern coefficients by using maximum likelihood fitting for Poisson distributed uncertainties. Coefficients for the basis patterns depend algebraically on dipole orientation.

The lateral PSF readily undergoes the 6 basis pattern devolution in 2-D because the CCD camera records the lateral photon distribution in the 2-D pixel array. The 2-D spatial pattern defines the dipole orientation and contains information specifying the axial position of the probe. Basis patterns depend on the axial dimension conferring sensitivity to the image for the axial position of the probe because a changing sample axial position alters pattern shape. We calibrated the axial dimension of image space to exploit the position sensitivity of the patterns using an axial translating camera. The setup records the axial photon distribution using the translating CCD detector. It provides <10 nm precision (super-resolution) of the peak position of a point object and calibrates the axial image space against the real axial dimension in sample space. When calibrated, we assign relative sample space axial distances to emission patterns. We also show that an axial scan data set consisting of three images from a translating objective on a single emitter provides superior resolution for all quantities investigated.

We tested the basis pattern analysis method for probe dipole orientation detection on photoactivated green fluorescent protein (PA-GFP) tagged myosin regulatory light chain (RLC) exchanged, permeabilized skeletal muscle fibers, in rigor. Orthogonal polarized photoactivation laser pulses were applied to the PA-GFP tagged myosin cross-bridges in separate fiber fields to photoselect contrasted oriented sub-populations of probes that are intrinsically oriented by fiber structure. Evidence for both orientation selecting processes seen in the data validates the approach.

## Methods

### Ethics Statement

Our protocol for obtaining rabbit tissue is approved by the Mayo Institutional Animal Care and Use Committee, Protocol A4208.

### Chemicals

ATP, dithiothreitol (DTT), leupeptin, and phenylmethanesulfonyl fluoride (PMSF) were from Sigma Chemical (St. Louis, MO). All chemicals were analytical-grade or Ultra-Pure if available. Carboxylate-modified fluorescent microspheres were purchased from Molecular Probes (Eugene, OR).

### Solutions

Rigor solution contains 10 mM imidazole, 2.5 mM ethylene glycol bis(β-aminoethyl ether)-N,N,N′,N′-tetraacetic acid (EGTA), 2.2 mM Mg acetate, 130 mM potassium propionate, 0.2 mM PMSF, 0.8 µg/mL leupeptin, and 1 mM DTT.

### Samples

Psoas muscle fibers were prepared from rabbit and the native myosin regulatory light chain (RLC) exchanged with PA-GFP tagged human cardiac RLC (HCRLC-PAGFP) as described [9], [10].

Red-orange carboxylate-modified fluorescent spheres had 40 nm diameter and excitation/emission maxima at 565/580. Sphere concentrations were computed using the formula from the manufacturer and diluted in water. We used a 10^4^ fold dilution from stock into distilled water giving sphere concentration of 1.4×10^11^ spheres/mL.

All experiments were conducted at room temperature.

### Sample Chamber

Clean #1 glass coverslips were sonicated for 10 min in ethanol then plasma cleaned (Harrick Plasma, Ithaca, NY) for 15–30 min. Coverslips were placed on a 1×3-inch brass slide with a large hole cut out, permitting the objective from the inverted microscope to contact the coverslip through immersion oil. A water tight chamber was constructed on top of the coverslip as described [9]. The chamber contained either a muscle fiber or a suspension of nanospheres.

Through-the-objective total internal reflection (TIR) occurs at the coverslip/aqueous interface where a HCRLC-PAGFP exchanged muscle fiber in aqueous buffer solution contacts the coverslip and is illuminated by the evanescent field. Diluted nanospheres were flowed into the sample chamber and allowed to dry. After drying, some spheres were strongly attached to the substrate surface in a sparse random spatial distribution that remained intact when distilled water refills the sample chamber. Fluorescent spheres were TIR or epi-illuminated depending on the experiment.

### Microscopy

**Figure 1:**
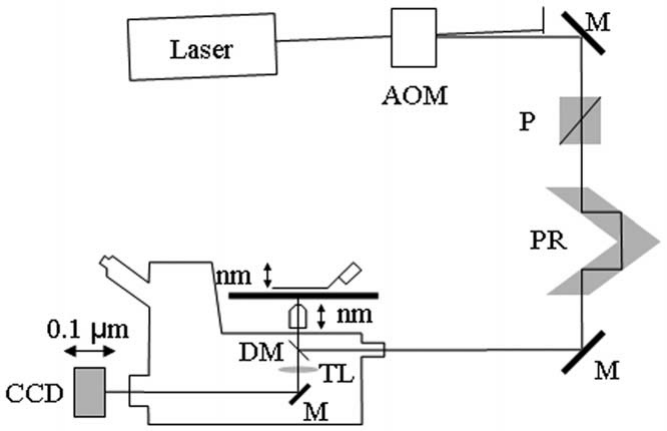
Inverted microscope (Olympus IX71) setup. Diagram shows excitation and emission detection pathways and double edge arrows indicating translating elements with their approximate spatial resolution. The 488 or 514 nm lines from the argon ion laser (Innova 300, Coherent, Santa Clara, CA) are intensity modulated by the acoustoptic modulator (AOM) then linearly polarized at the Glan-Taylor (P) polarizer. The polarization rotator (PR) uses Fresnel Rhombs to rotate linear polarized light to the desired orientation. The beam enters the microscope, reflects at the dichroic mirror (DM), and is focused on the sample by the objective. The high NA objective (Olympus planapo 60X, NA = 1.45 or TIRF objective) translates along the optical axis under manual control using the microscope focus or with nm precision using a piezo nanopositioner (C-Focus, MCL, Madison, WI). The C-focus translates the objective under computer control and has an alternative feedback mode where it maintains a constant distance between the objective and a set point on the microscope stage. Sphere samples were sometimes mounted on a piezo stage to alter the distance from sample to objective along the optical-axis with nanometer precision when a moving objective was undesirable. Emitted light is collected by the objective, transmitted by the dichroic mirror, then focused by the tube lens (TL) onto the CCD camera with 6.45 µm square pixels (Orca ER, Hamamatsu Photonics, Hamamatsu-City, Japan). In some experiments, a microscope stage with leadscrew drives and stepper motors translate the CCD camera with submicrometer resolution (LEP, Hawthorne, NY).

Figure 1 shows the microscope (Olympus IX71) with excitation and emission detection pathways. Double edge arrows indicate translating elements in the apparatus with their approximate spatial resolution. Laser lines are intensity modulated by the acoustoptic modulator (AOM) then linearly polarized at the polarizer (P). The polarization rotator (PR) performs the final polarization adjustment before entering the microscope. The objective translates along the optical axis under manual control using the microscope focus or with nm precision using a piezo nanopositioner. Sphere samples adsorbed to a cover glass were sometimes translated on a piezo stage to alter the distance from sample to objective along the optical axis with nanometer precision when a moving objective was undesirable (nanopositioners from MCL, Madison, WI). Emitted light is collected by the objective, transmitted by the dichroic mirror (DM), then focused by the tube lens (TL) onto the camera. In some experiments, a microscope stage with leadscrew drives and stepper motors (LEP, Hawthorne, NY) translate the camera.

Overall computer control of the microscope is exercised through a custom written Labview (National Instruments, Austin TX) routine and drivers supplied by the manufacturers. The Labview software coordinates image capture by the camera with movement of the various translating elements in or around the microscope. Translating elements were controlled via a RS232 port (LEP stage) or a USB interface (MCL nanopositioners). Wasabi! software (Hamamatsu Photonics, Hamamatsu-City, Japan) captures images following triggering by the Labview program via a counter output TTL pulse (NI6602).

For TIRF microscopy, laser excitation is focused on the back focal plane of the objective and incident from the glass side of a glass/aqueous interface at angles greater than critical angle for TIR. Although light is totally reflected, an evanescent field created in the water medium and decaying exponentially with distance from the interface, excites fluorophores within ∼100 nm of the surface [11]. P-polarized incident light has electric field polarization in the incidence plane and produces an elliptically polarized evanescent electric field. Evanescent intensity is predominantly polarized normal to the interface [12]. S-polarized incident light has electric field polarization perpendicular to the incidence plane that is continuous across the interface. For a muscle fiber, 7 (parallel) or ⊥ (perpendicular) means relative to the fiber symmetry axis. The incident beam always propagates along the interface in a direction perpendicular to the fiber symmetry axis hence p(s)-polarized incident TIR light produced the ⊥(7) excitation.

A HCRLC-PAGFP exchanged muscle fiber is illuminated by the evanescent field. Sparse PA-GFP photoactivation was accomplished under TIR using 10–20 sec exposure to 488 nm light from the argon ion laser. Laser intensity was ∼10–100 fold higher during photoactivation compared to that used during fluorescence excitation of photoactivated PA-GFPs. A larger field of the muscle fiber was shown previously showing the sparse photoactivation of the PA-GFP under these conditions [1], [10]. Photoactivated PA-GFPs in the muscle fiber are apparently single molecules because their density in the fiber image implies infinitesimal probability for two photoactivated molecules to reside in one pixel.

### Photoselection of Oriented Photoactivated Chromophores

Irreversible isomerization, N_B_→N_A_, where total molecules N is the sum of un-photoactivated (N_B_) and photoactivated (N_A_) species, describes fluorescence photoactivation. Solving for N_A_(t),
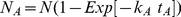
(1)where k_A_ is the activation rate and t_A_ is the activating light pulse duration. Integrated absorption cross-section, k_A_ ∝ 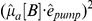, where 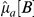 is the absorption dipole moment for the un-photoactivated species (wavelength band near 400 nm) and 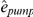 is the photoactivating (pump) light electric field polarization vector. For single molecule *i*, the normalized probability for its photoactivation, γ_A,i_, is, 
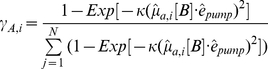
(2)where κ is a constant dependent on activating light pulse duration and other factors excluding probe dipole orientation. In all experiments κ is small to ensure that a sparse population of probes is photoactivated and to achieve the most selective orientation distribution of photoactivated probes. Photoactivation is a rare event with γ_A,i_ << 1. In simulation a random number, ξ, between 0 and 1 is compared to γ_A,i_. When ξ < γ_A,i_, molecule *i* is photoactivated. This procedure selects with higher probability molecules having 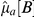 parallel to 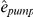.

For comparison with results reporting polarized emission intensity ratios, we compute the photoactivated single molecule polarized fluorescence, F_i,e,ν_, 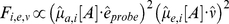
(3)where 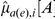 is the absorption (emission) dipole moment for the i^th^ molecule of the photoactivated species (wavelength band near 490 nm), 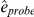 the unit vector in the direction of the exciting (probe) field, and 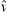 is the emission polarizer orientation. Fluorescence polarization ratios are defined,
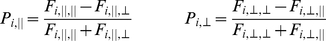
(4)where 7 or ⊥ means relative to the fiber symmetry axis. Because ratios in eq. 4 refer to single photoactivated molecules, the dependence on 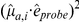 (or the second index on F) cancels and does not contribute to the ratio value. All excitation photoselection is accomplished with the polarized photoactivation that is dependent on 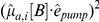
(eq. 2). Mixed illumination polarization ratios such as Q_||_  =  (F_||,||_ − F_⊥,||_)/(F_||,||_ + F_⊥,||_) will contain dependence on 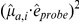.

Simulation of the photoselected set of activatable probes allows separate orientations for 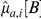 and 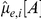. Photoactivated PA-GFP dipole moments 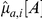 and 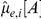 have ∼0.3 limiting anisotropy corresponding to an angle of ∼24° between dipoles [10]. Comparison of simulation and measurement suggests the angle between 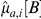 and 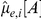 is larger (see RESULTS).

### Calibration of Image Space Axial Dimension

**Figure 2:**
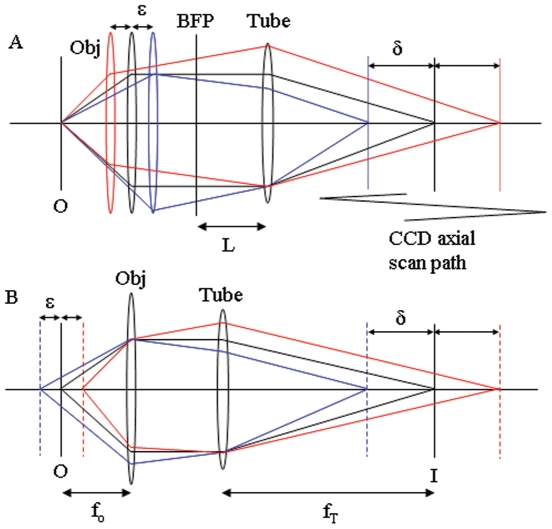
Ray diagram for object and image axial positions. Object displacement ε from the objective effective focal point at O gives image displacement δ from the tube lens focal point at I. The CCD axial scan path shows camera translation in image space. BFP is the back focal plane of the objective and L is the tube length. The Tube lens has focal length f_T_. Panel A shows point source repositioning relative to a fixed microscope stage due to translation of the objective (Obj) with focal length f_O_. Panel B shows an equivalent point source repositioning due to a translating microscope stage.

The linear relationship between object and image dimensions in lateral coordinates is the objective magnification. The analogous relationship for the axial dimension was formulated from the lens equations. Figure 2 shows the relationship of axial object and image positions for object displacement ε from the objective effective focal point and image displacement δ from the tube lens focal point. Solving the coupled lens equations for this system we obtain,
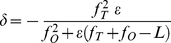
(5)where f_O_ is the objective effective focal length, f_T_ is the tube lens focal length, and L is tube length. In all measurements, ε≤1 µm, while other quantities in eq. 5 are 3–180 mm hence δ and ε are linearly related with axial magnification, M_a_, given by the relationship *n*M_a_ ≈ −(f_T_/f_O_)^2^ for *n* the sample space refractive index. The negative sign in eq. 5 indicates that when the sample moves away from the objective the image moves towards the tube lens. Parameters appropriate for the Olympus IX71 and TIRF objective (Olympus planapo 60X, NA = 1.45) have f_T_ = 180 mm, f_O_ = 3.0 mm, and L ∼150 mm indicating *n*M_a_ ≈ −3600. Projective transformation has *n*M_a_ = −M_L_^2^ where M_L_ is the lateral magnification likewise suggesting *n*M_a_ = −3600 [13].

The axial PSF observed from fluorescent sphere point source at various distances from the objective are compared to the theoretical PSF to estimate the mean sphere axial position. We describe how to compute the theoretical PSF in RESULTS. The point source is localized to higher precision than the diffraction resolution limit by fitting the calculated PSF to its measured photon distribution. We estimated precision by the criterion derived by Bobroff [2] using variance, σ^2^, that is the sum of variances from camera and signal noises, σ_c_^2^ and σ_s_^2^, such that,
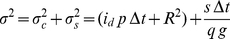
(6)for i_d_ the dark current in electrons/(pixel-sec), p the number of pixels in the array containing the in-focus point source image, Δt the collection time interval for a single image capture, R the rms read out noise, *s* above-background signal in electrons/sec, q the CCD quantum efficiency, and g the CCD gain. SNR is *s*/σ. ORCA CCD camera specifications have i_d_  = 0.1 electrons/(pixel-sec), R = 6, and q = 0.7 for light at 550 nm wavelength. We utilized p = 4 or 9, Δt = 500 ms, and g = 1. A camera scan pathway shown in Figure 2 covers 6–12 mm in image space in ∼40 sec.

**Figure 3:**
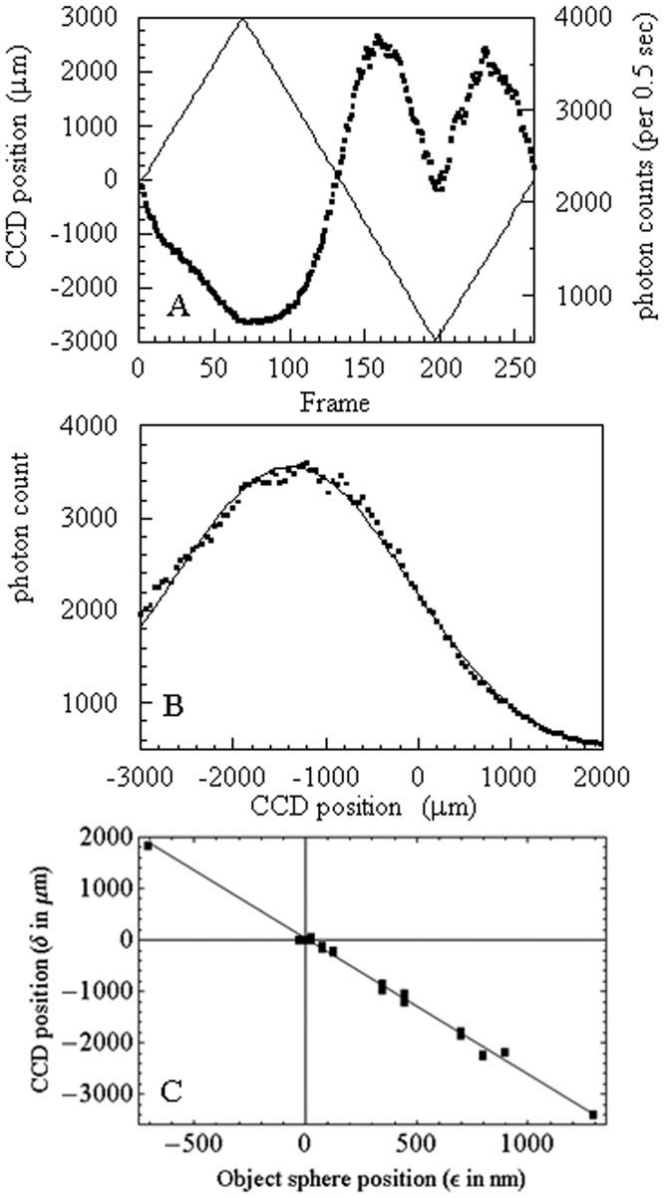
A translating CCD camera records the axial PSF to calibrate axial image space. Panel A shows the camera position saw-tooth pattern (solid line) and the fluorescence intensity observed from the point source (▪). The left hand side abscissa scale applies to the saw-tooth curve. The right hand side abscissa scale applies to intensity (▪). Intensity is the sum of photons in 2×2 or 3×3 pixel regions defining the focused point source. Panel B is the camera axial position (independent variable) vs fluorescence intensity (dependent variable) including only the middle portion of the saw-tooth pattern where camera position changes monotonically. The fitted curve is the PSF computed as described in RESULTS. Panel C shows the calibration curve indicating the relationship between δ and ε in Figure 2. The slope of the curve is the axial magnification, M_a_.

Axial camera scanning was usually slightly off axis such that the image from the point-object moved laterally in the camera field of view. Slightly off-axis camera scanning does not affect calibration because lateral movement is negligible compared to axial movement. Images were aligned by requiring maximal intensity overlap between the in-focus point-object image frame and the other frames in the scan. Best results were obtained when several point-objects covered the field of view and multiple sources were optimally aligned simultaneously. **Figure 3** **panels A** and **B** show measured emission axial intensity profile from a fluorescent point source fixed on the coverslip under epi-illumination. Experiments were conducted by focusing the objective on a fluorescent sphere specimen then translating the CCD camera axially through the saw-tooth pattern indicated. Axial translation sweeps the camera through the point source image space recording the axial PSF. Total photon count at each axial position is the sum of counts in the lateral pixels occupied by the focused point object image. Panel A shows the camera position saw-tooth pattern (solid line) and the fluorescence intensity observed from the point source (▪). The left hand side abscissa scale applies to the saw-tooth curve. The right hand side abscissa scale applies to intensity (▪). In Panel B, the camera axial position (independent variable) vs fluorescence intensity (dependent variable) includes only the middle portion of the saw-tooth pattern where camera position changes monotonically. We computed the fitted curve in Panel B (solid line) as described in RESULTS.

Figure 3 panel C calibrates axial image space by comparing point source displacement (ordinate) read from the nanopositioner with the observed position derived from the mean photon distribution in the axial PSF (▪). The best fitting curve produces the slope, M_a_ = −2644±42, in agreement with the lens equation estimate giving M_a_ ≈ −3600/1.334 = 2699 for *n* = 1.334 (refractive index of water in sample space). Experiments were conducted as in Figure 3 for several sample or objective positions. Data points are derived from epi- or TIRF illumination where point source nanopositioning relative to the fixed microscope stage occurs due to translation of the objective (Figure 2A) or translation of the point source (Figure 2B), respectively.

Precision, indicated in Figure 3C by error bars that are smaller than the solid square symbols, is 2–9 nm after conversion (including error propagation) from μm. The observed position twice differs from the fitted line by more than precision estimates at 800 and 900 nm suggesting measurement accuracy error is somewhat larger than precision. Accuracy error is probably due to objective drift inherent in the Olympus IX71 stand.

## Results

### Orientation and Axial Spatial Dependence of Image Space Intensity

The electric field at the camera image plane was computed for the TIRF microscope described above using a method taken from Richards and Wolf [14]. We propagated the polarized emission through the objective starting from the intensities transmitting the glass coverslip (separating objective and sample) for a dipole, 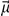, near an interface as derived by Hellen and Axelrod [5]. They defined what we call the TIRF-coordinates with z-axis normal to the glass/aqueous interface pointing into the aqueous phase, x- and y-axis in the plane of the interface and perpendicular (x-) or parallel (y-) to the fiber symmetry axis. We assume all dipoles are 50 nm from the interface in the aqueous medium implying that the dipolar emission field propagating into the glass medium is significantly perturbed according to the rules derived in [5]. The electric field in the glass medium before entering the objective, 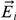, is expressed relative to unit vectors, 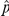, 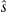, and 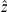 defined by an observation plane comprised of the observation point and the z-axis such that 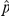 lies in the observation- and interface-planes, 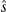
lies in the interface plane but perpendicular to the observation plane, and 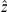
along the z-axis. Then,
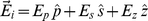
(7)for field amplitudes given in eq. 33 from [5]. Orientation of the {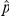,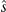,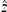} coordinate system also defines the meridional plane that is equivalent to the observation plane.

The electric field approximately conserves its polarization relative to the meridional plane after refraction in the objective [14]. Axelrod's A-matrix, defined for this purpose, rotates the incoming electric field polarization due to refraction at a lens while conserving the angle the field vector makes with the meridional plane [6]. The A-matrix is the Euler rotation A_1_ = Eu(φ,θ-π,-φ) for,
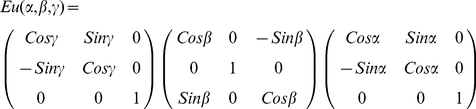
(8)for θ and φ the polar and azimuthal angles of incoming plane waves in the meridional plane relative to fixed TIRF coordinates. Refracted emission, A_1_ 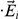, propagates in the negative direction along the z-axis. It is more convenient to change coordinates to have the emitted field propagating in the +z direction accomplished by a rotation through π and about x-axis where,
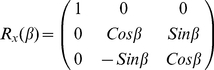
(9)


such that 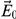 = R_x_(π).A_1_ 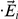. The new fixed coordinates are lab-coordinates derived from TIRF-coordinates using R_x_.


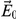
 emerges from the back aperture where it impinges on the DM and barrier filter (barrier filter not shown in Figure 1). The DM is mounted at 45° to the propagating light direction and introduces an amplitude change and phase shift to the transmitted light field components via complex transmission coefficients, t_p_ and t_s_. We assume the DM contains a dielectric thin film interface with multiple interfering reflections to give the DM the ability to reflect and transmit the desired light wavelengths. We adjusted film thickness parameters in t_p_ and t_s_ to give relative intensities consistent with a correction factor measured from a sample of rapidly tumbling chromophore emitters in solution. The rapidly tumbling chromophores emit unpolarized light that is linearly polarized before the DM by introduction of an analyzer. The tumbling probe and analyzer provide linearly polarized light of uniform intensity for any direction selected by the analyzer orientation. Polarized light intensity collected with analyzer along the x-axis compared to that collected with analyzer along the y-axis gives the ratio |t_p_|^2^/|t_s_|^2^ ≈ 1.4. Transmission coefficients multiply the incident field with the x- and z- polarization components multiplied by t_p_ and the y-component by t_s_ given in matrix form by,



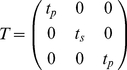
(10)The polarized sample fluorescence transmitting the DM,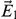 = T 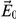, separates into three complex vectors multiplying the x-, y-, and z- components of 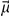 such that,
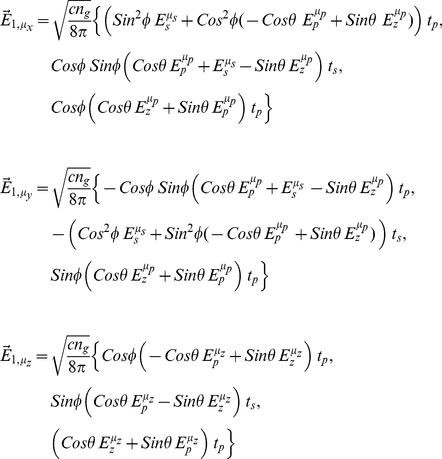
(11)where c is the speed of light, n_g_ is the refractive index of glass, field amplitudes 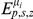 are from eqs. 26–28 in [5] and depend on θ but not φ. Another A-matrix, A_2_ = Eu(φ,−θ_T_,−φ), rotates the electric field polarization due to refraction at the tube lens giving the electric field at the tube lens focus, 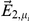 = A_2_ 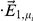. Angle φ is identical to that in A_1_ while,



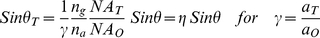
(12)defines θ_T_ from θ where *n_a_* is the refractive index of air, NA_T(A)_ the numerical aperture of the tube (objective) lens, and γ the ratio of aperture radii for the tube and objective lenses. Constant *η* for our microscope is ∼0.01 hence θ_T_ ranges over a smaller interval than θ implying the correction from A_2_ is much less significant than that from A_1_.

**Figure 4:**
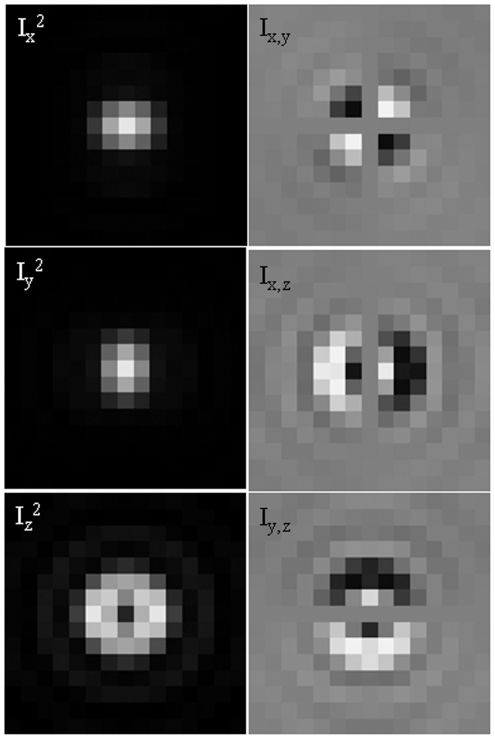
Dipole emission basis patterns. Resolution shown is appropriate for the Olympus IX71, the 60X TIRF objective, and 6.45 µm square pixels. Patterns in the left column depict intensities and are always ≥0. Patterns in the right column have negative values depicted as darker than regions around the edge where values are zero. Positive pattern values are brighter than edge values. Subscripts on I represent the dipole moment components contributing.

Time-averaged fluorescence intensity observed from individual molecules,



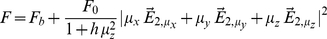
(13)where F_b_ is background, F_0_ the signal amplitude, 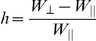 for W_‖,⊥_ the total power emitted by a dipole oriented parallel (with μ_z_ = 0) or perpendicular (with μ_z_ = 1) to the dielectric interface, and μ_i_ the single chromophore dipole components in lab-coordinates. In our application, *h* ∼ 0.12. Re-expressing eq. 13 in real quantities,



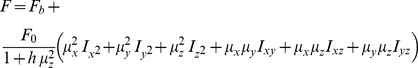
(14)for,
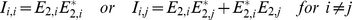
(15)The 6 “intensities” defined in eq. 14 are basis patterns spanning the 3-D image space for a dipole emitter, however, only the quantities 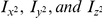 are observed independently. Other basis patterns have negative regions that are constrained to combine linearly with positive regions from other basis patterns to form the observed light intensity. Normalized basis patterns in 2-D (lateral dimensions) are shown in Figure 4 with 8 bit intensity resolution for axial dimension at the nominal focus. Negative “intensity” in the right column patterns (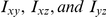) is depicted as darker than regions around the edge of the pattern where intensity is zero. Cylindrical symmetry expected for the 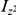 pattern is broken by the presence of the DM that preferentially transmits light polarized along the x-axis.

**Figure 5:**
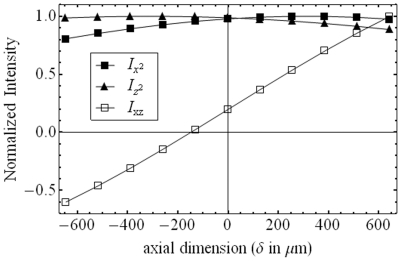
Normalized intensity axial dependency. Intensities 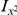, 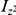 and 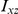 from eqs. 14 & 15 show peaks for 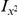
and 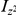
occur at different axial positions.

Figure 5 shows the axial dependencies in image space for 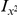, 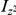 and 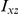 (curves for 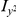 and 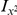 are identical). Peak intensities for 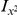 and 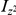 occur at different axial positions (compare peak shapes at zero in the axial dimension). This condition is amplified by the presence of the DM but occurs to a lesser extent without it. Patterns 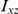 and 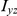 (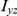 not shown) vary over most of their amplitude over the axial domain indicated in Figure 5 and over large regions of the image plane. The intensity gradient across patterns confers position sensitivity because a changing sample axial position alters pattern shape. We calibrated the image space axial dimension using the fluorescent nanospheres observed with the axially translating camera described in METHODS. When calibrated, we can exploit the position sensitivity of the patterns to assign relative sample space distances to emission patterns.

### Pattern Recognition

Substituting 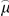  =  (Sinθ_p_Cosφ_p_,Sinθ_p_Sinφ_p_,Cosθ_p_) into eq. 14 and rearranging terms to find,
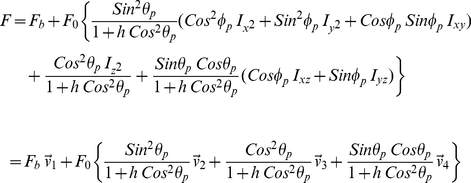
(16)we condense the pattern spanning basis to the four φ_p_–dependent vectors 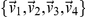 as defined above and solve for their respective coefficients using the generalized linear model (GLM), maximum likelihood fitting, for Poisson distributed uncertainties. This fitting method was demonstrated to be better for treating low signal level photon counting data than χ^2^ minimization [15], [16]. In our fitting routine, θ_p_ and φ_p_ are sampled over the interval {0,π} since dipole inversion symmetry implies it covers all of sample space. We sampled axial distance ε over the {−250,250} nm interval since it extends beyond the ∼100 nm evanescent illumination depth used in experiments on muscle fibers. With each choice of φ_p_, GLM fitting finds the coefficients for 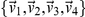 that are the maximum likelihood fit for one observed single molecule fluorescence pattern. After densely sampling φ_p_ we choose the maximum of all the maximum likelihood fits to select the best φ_p_ and coefficients of 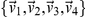 then compute from them the background light intensity level, F_b_, signal intensity, F_0_, and angle θ_p_. The fluorescence pattern determines (θ_p_,φ_p_) up to the emission dipole inversion symmetry (π−θ_p_,π+φ_p_).

### Accuracy of Pattern Recognition Tested in Simulated Data

**Figure 6:**
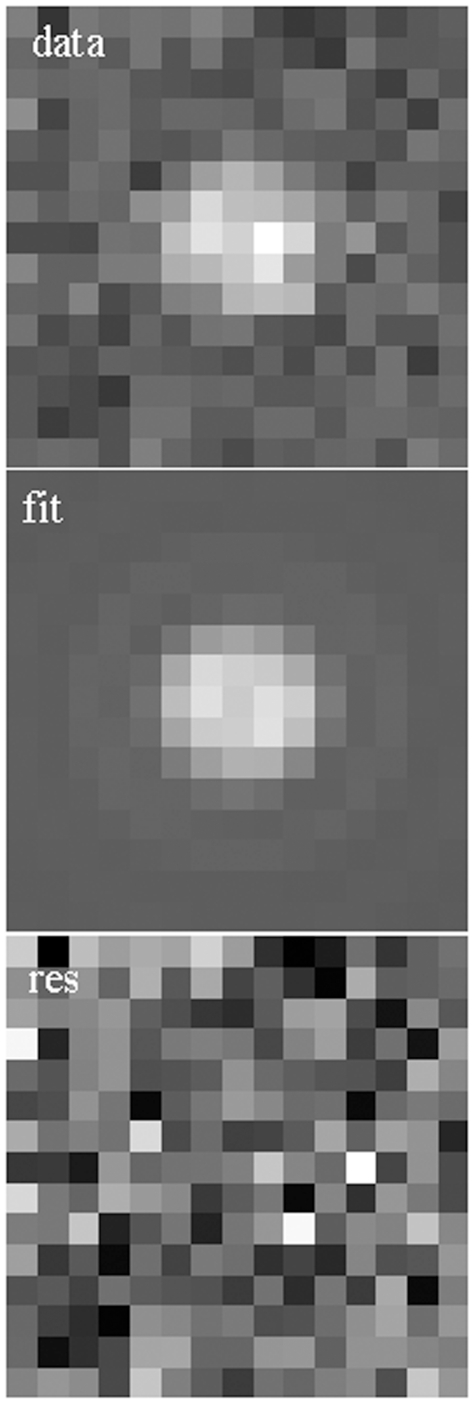
Simulated fluorescence emission pattern for a dipole with polar and azimuthal angles (θ_p_,φ_p_)  =  (28.8,153). Background fluorescence and camera noise contribute to the Poisson distributed noise of the total signal (top panel, *data*). The fitted pattern (middle panel, *fit*) was identified by the GLM, maximum likelihood fitting, for Poisson distributed uncertainties. The residual of the two patterns normalized to fill the 8-bit dynamic range is shown (bottom panel, *res*).

**Figure 7:**
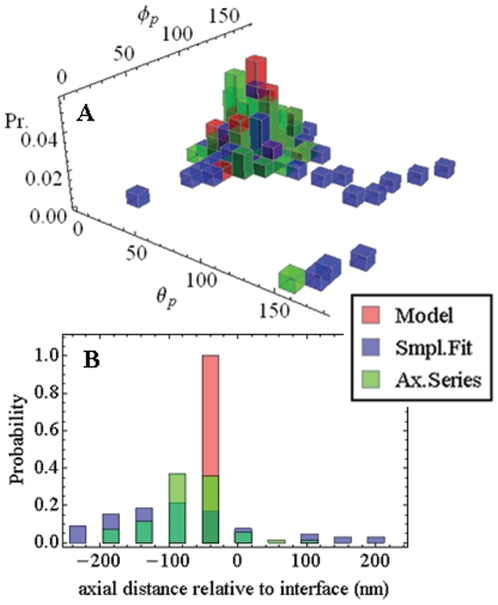
Model orientation distribution and its representation obtained by different data fitting methods. Panel A. Orientation distribution for the model (red) with normally distributed dipole polar and azimuthal angles (θ_p_,φ_p_) covering a 15 degree width with average values (<θ_p_>, <φ_p_>)  =  (45,120) degrees (Model). The sample set contains 70, (θ_p_,φ_p_), pairs. Shown in blue is the orientation distribution corresponding to the model but obtained by fitting individual single molecule fluorescence patterns generated from the (θ_p_,φ_p_) pairs (Smpl. Fit). Depicted in green is the orientation distribution corresponding to the model data but obtained by simultaneously fitting single molecule fluorescence patterns in groups of three from an axial scan series (Ax. Series). Panel B shows the axial distributions for the model (a red single spike at −50 nm), by fitting single molecule fluorescence patterns (blue), and by simultaneously fitting single molecule fluorescence patterns in groups of three from an axial scan series (green).

We generated simulated dipole emission patterns for normally distributed polar and azimuthal lab-coordinate dipole orientation angles (θ_p_,φ_p_). Several combinations of normally distributed angles and various axial dipole positions were investigated. We report here on normally distributed angles covering a 15 degree width with average values (<θ_p_>, <φ_p_>)  =  (45,120) degrees and with dipoles positioned axially at −50 nm corresponding to a dipole in the aqueous phase and 50 nm above the glass/aqueous interface. Signal fluorescence, F_0_, and background, F_b_, (eqs. 6 and 14) from background light and camera noise are 143 and 35 and similar to typical muscle fiber data. Quantities were substituted into eq. 16 to generate an ideal pattern then each pixel intensity was Poisson distributed. Figure 6 shows a simulated pattern for (θ_p_,φ_p_)  =  (28.8,153) with Poisson distributed noise (top panel, *data*), the fitted pattern (middle panel, *fit*), and the residual of the two patterns normalized to fill the 8-bit dynamic range (bottom panel, *res*). The fit gave (θ_p_,φ_p_)  =  (33.1,130) and the axial position ε = −50 nm. Figure 7 shows the orientation distribution (panel A) and the axial distribution (panel B) for the model (red) and fitted data (blue) derived from 70 simulated patterns. The fitted simulated data derived orientation distribution accurately represents the normal model distribution except for the occasional outlier that we traced to a misreading of the coefficient for the basis pattern ν_4_, c_4_. The c_4_ sets the sign for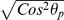 that is inverted to solve for θ_p_. The wrong sign converts the correct θ_p_ to its complement π−θ_p_ as can be seen in 8 cases out of the 70 shown in Figure 7. These outliers occur when θ_p_ ≈ 0, 90, or 180 degrees when c_4_ is close to zero and parameter standard error (determined from the covariance matrix) is > |c_4_|. Rising signal-to-noise (S/N given by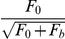) gradually eliminates the erroneous assignments except when c_4_ = 0. Then the sign of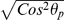 is irrelevant or can not be determined due to dipole inversion symmetry.

The fitted simulated data derived axial distribution does not correctly identify axial dipole position in each pattern (the model distribution in this case is a delta function at ε = −50 nm), however, the mean axial position of the dipole is −60 nm. The results probably reflect different sensitivities of basis patterns for axial position. Dipole orientations containing larger contributions from I_xz_ and I_yz_ will be inherently more sensitive to dipole axial position since these patterns have a larger “intensity” gradient in the axial dimension near the nominal focus.

A different approach was tested in simulation that shows promise. Simulated images from an axial scan of the single molecule emitters produced data like that used above plus images from above and below the nominal focal plane. The axial scanned images were fitted simultaneously to constrain the dipole orientation degrees of freedom. The scan consisted of three images of the emitter at −100, −50, and 0 nm replacing the single image at −50 nm discussed above. Scanning does not change dipole distance from the glass/aqueous interface but the objective is moved these distances in the axial dimension (in practice, using the nanoposititoner shown in Figure 1). The additional information provided by the multiple images removes all but one of the outliers due to the incorrect assignment of c_4_. Figure 7 Panels A and B show results (green) for the axial scanned data analysis.

### HCRLC-PAGFP Exchanged Muscle Fibers in Rigor

**Figure 8:**
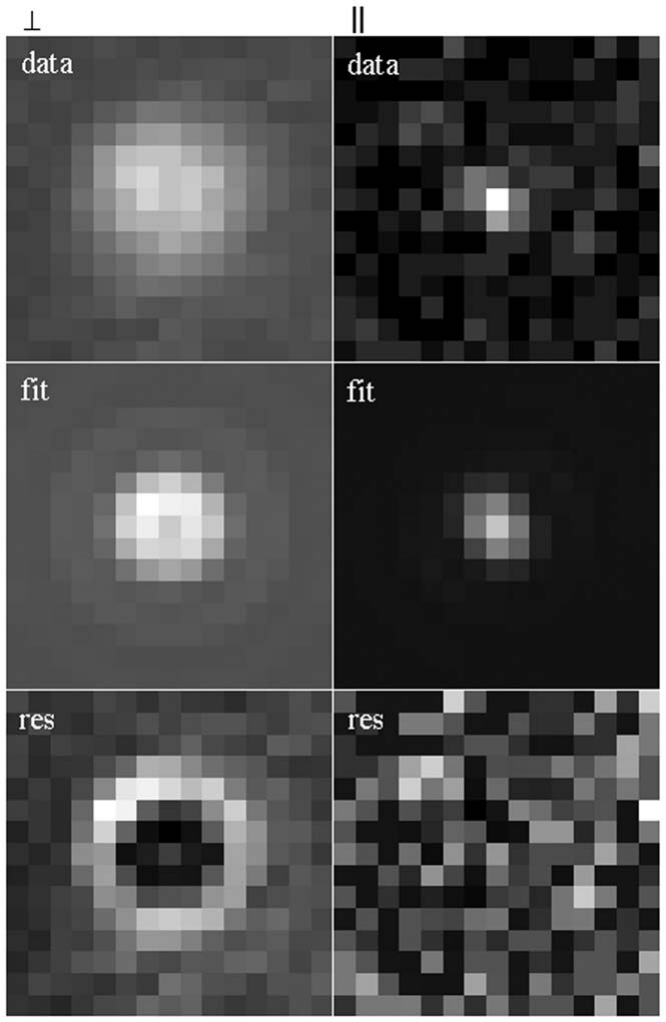
Single molecule data from muscle fibers. Single PA-GFP tagged cross-bridges from fibers in rigor from the perpendicular (left) and parallel (right) polarization photoactivated subset. The top images are measured data, middle images fitted data, and bottom images the residuals.

Single molecule data from PA-GFP tagged myosin cross-bridges in permeabilized muscle fibers was collected from several fiber samples over several days. PA-GFP photoactivation with light polarized parallel or perpendicular to the fiber axis photo-induced an ordered subset of single probes within a differently ordered set of cross-bridges. Myosin cross-bridges are intrinsically orientationally ordered due to the fiber structure. Figure 8 shows single PA-GFP tagged cross-bridges in rigor from the perpendicular polarization photoactivated subset (top left, *data*) and the parallel polarization photoactivated subset (top right, *data*). The middle panels are the fit to the data (*fit*) and the bottom panels the residual normalized to fill the 8-bit dynamic range (*res*). The perpendicular polarization photoactivated pattern has the characteristic donut shape of a dipole (the emission dipole of the photoactivated species or μ_e_[A]) perpendicular to the coverslip/aqueous interface. In Lab-coordinates where 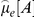  =  (Sinθ_p_Cosφ_p_,Sinθ_p_Sinφ_p_,Cosθ_p_) this particular dipole has (θ_p_,φ_p_)  =  (143,100) in degrees. The residual shows that the fit is less sprawling than data suggesting the image is somewhat unfocused. The parallel polarization photoactivated pattern has the filled in and more compact shape of a dipole parallel to the coverslip/aqueous interface. In Lab-coordinates it has (θ_p_,φ_p_)  =  (92,120). Perpendicular and parallel polarization photoactivation tends to select cross-bridges perpendicular and parallel to the fiber axis. Transforming (θ_p_,φ_p_) to the Fiber-coordinates azimuthal and polar angles (α,β), that specify the Euler angles for the GFP emission dipole, we find (97,55) and (175,30) for perpendicular and parallel photoactivation.

**Figure 9:**
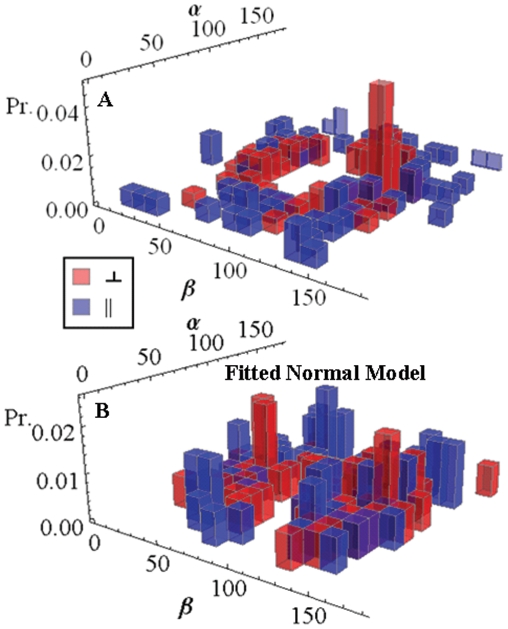
Orientation distribution probability histograms. Panel A: The orientation distribution for 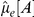 in fiber-coordinates (α,β) for perpendicular polarized photoactivation (red) and parallel polarized photoactivation (blue) detected from PA-GFP tagged muscle fibers in rigor. Panel B: The orientation distribution for 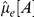 in fiber-coordinates derived from simulated data from the model distribution in eq. 17 for β_0_ = 47, σ_β_ = 20, γ_B,0_ = 0, and σ_γ_ = 1 degrees. Simulated data was fitted by the pattern recognition method used to fit the muscle fiber data shown in Panel A.

79 and 70 patterns like those in Figure 8, for perpendicular and parallel polarization photoactivation, were fitted and the data summarized in a 3-D histogram in (α,β) (Figure 9A). The data is plotted with the vertical axis showing probability (Pr.) rather than single molecule events to normalize the two data sets. The perpendicular polarization photoactivated (red) population is localized to regions where β ≈ 45 and 135 while α ≈ 90 and 270 degrees (regions where α>180 degrees correspond to the inversion symmetry peak not shown in Figure 9A) while the parallel polarization photoactivated (blue) population is more evenly distributed in both α and β. Considering the α-degree of freedom first, perpendicular polarization activates probes along the Fiber-coordinates y-axis since this axis is perpendicular to the coverslip/aqueous interface where α = 90 or 270 degrees. Parallel polarization activates a uniform distribution in α by symmetry. The underlying fiber azimuthal symmetry is consistent with the observations since probe ordering (in the α-degree of freedom) is then defined exclusively by photoactivation for perpendicular polarization and is unaffected by parallel polarization.

In the β degree of freedom, intrinsic fiber ordering also contributes to the observed probe distribution. This is clear in perpendicular polarization photoactivation (Figure 9A) since the predominant angles are 45, 135 degrees while the photoactivating field polarization is 90 degrees. We further investigated probe angular distribution in the fiber with the help of a model describing intrinsic ordering of the dipoles imposed by fiber structure. Euler angles (α,β,γ_B_)_i_ specify 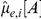 along the z-axis of a GFP fixed coordinate system (probe frame) and the un-photoactivated probe absorption dipole, 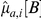, lying in the xz-plane of the probe frame at angle χ_B_ from 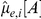 [10]. Orientation of 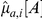 sets the absolute intensity of the emission pattern and is irrelevant for our single molecule analysis. We surmise χ_B_ from fluorescence polarization anisotropy with 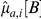 absorption and 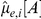 emission in un-photoactivated HCRLC-PAGFP. The 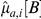 absorption is excited exclusively in un-photoactivated HCRLC-PAGFP at 400 nm and fluorescence polarization anisotropy indicates χ_B_ δ24 degrees (see Figure 2 in [10]). A Normal distribution of probe frames models fiber intrinsic ordering with means, β_0_ and γ_B,0_, and standard deviation, σ_β_ and σ_γ_, where,
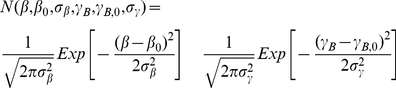
(17)


No dependence on α in eq. 17 implies the azimuthal symmetry assumed for the myosin and GFP probe distribution in the fiber.

We generated simulated data from the model distribution, fitted it by the pattern recognition method used to fit real data, then computed the orientation distribution for 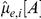 indicated in Figure 9B. Better agreement between simulated and observed 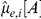 orientation distributions suggested that β_0_ = 47 and σ_β_ = 20 degrees, γ_B_ is narrowly distributed about γ_B,0_ ≈ 0, while χ_B_ is statically disordered. We statically distributed χ_B_ while constraining average anisotropy using, 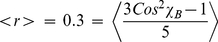
(18)


We found the normal distribution for χ_B_ with <χ_B_ > = 8 and width of 25 degrees provide a reasonable approximation to observations. Comparison of Figures 9A and 9B indicates the model data captures the main features of the probe orientation distribution with the perpendicular photoactivated population (red) broadly localized to α ≈ 90, 270 and β ≈ 45, 135 degrees while the parallel polarization photoactivated population (blue) is more evenly distributed over the (α,β) domain.

**Figure 10:**
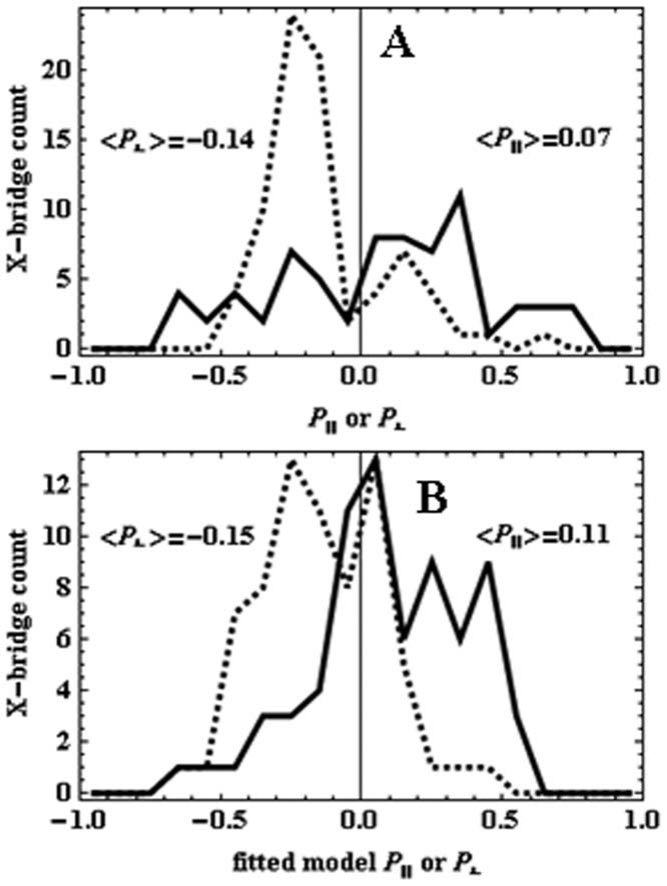
Polarization ratio histograms. Polarization ratios derived from the real and simulated data in Figure 9 with dashed lines indicating P_⊥_ and solid lines P_7_. Panel A: Polarization ratios derived from PA-GFP tagged muscle fibers in rigor from Figure 9A. Panel B: Polarization ratios derived from the simulated data in Figure 9B.

The orientation distribution of μ_e,i_[A] readily converts to the polarization ratios in eq. 4 and are shown in Figure 10 for observed (Panel A) and simulated data (Panel B). These data are in qualitative agreement with each other and with data published previously on the system where the polarization ratios were directly measured with conventional fluorescence polarization methods (see Figure 6 in [10]). Previous work had P_7_ and P_⊥_ displaced further from zero compared to present results suggesting a systematic difference from the correction factor (eq. 10). The correction factor is handled differently in the two methods. In the past it changed intensity of half the data sets uniformly while in the pattern recognition method it influences a fit applied independently to each single molecule intensity pattern. In the present study fluorescence patterns from fewer single molecules were quantified due to the more extensive analysis needed for each pattern.

**Figure 11:**
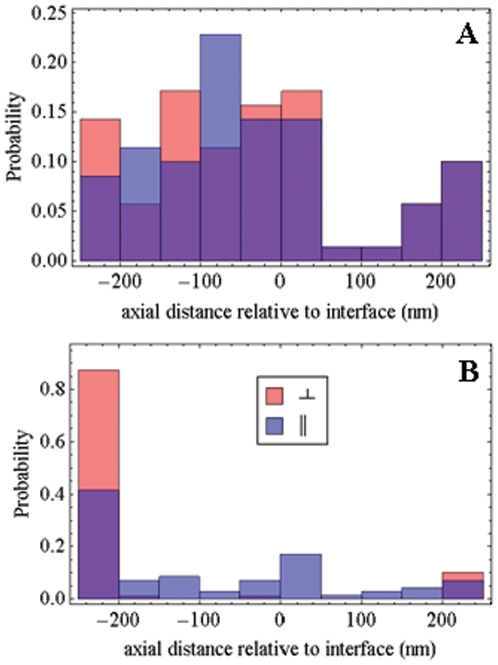
The probe axial spatial distribution probability histograms. Probe axial spatial distribution from simulated data (Panel A) and data detected from PA-GFP tagged muscle fibers in rigor (Panel B). Simulated data is the same as that used in Figure 9B. Muscle fiber data is the same as that used in Figures 8, 9, 10.

The emission dipole axial position is also quantified in the pattern fitting method. This is demonstrated in Figure 11 by the simulated data (from Figure 9B and Figure 10B) where patterns had dipoles in the water medium and positioned axially 50 nm from the glass/aqueous interface (−50 nm in the axial coordinate plotted). Figure 11A indicates the pattern fitting method locates the dipole position in the simulated data (mean axial distances of −47 and −46 nm for ⊥ and 7 polarized photoactivated probes) but did not do so for every pattern. Real data axial positioning, Figure 11B, indicates unresolved dipole positions at or beyond the arbitrary axial limit of our calculated emission pattern suggesting that either the human judged focus on the single molecule sample is incorrect or that the fitting method was hindered by practical uncertainties like a spatially uneven background. Regarding the former, sprawling point source data in Figure 8 resembles a slightly out of focus sample, and, regarding the latter, simulated data had Poisson distributed background light based on a uniform background average (conditions for the simulations were described in the previous section). Probability density for the parallel or perpendicular polarization photoactivated samples indicates ∼60% or ∼15% of the dipoles are within the arbitrary axial limit imposed by the emission pattern calculation. This suggests the parallel polarization photoactivated sample is easier to focus because the single molecule images are more point like. The parallel polarization photoactivated sample had a mean axial distances of −98 nm.

## Discussion

Point objects viewed in a microscope have the objective PSF defining the narrowest photon distribution in 3-dimensions detectable by the instrument. The objective PSF correlated with the measured sample photon distribution identifies the distribution center with precision determined by S/N ratio rather than the PSF distribution width [2], [3]. In super-resolution microscopy, the precise distribution center replaces the point object image providing a boost in spatial resolution [17], [18]. Lateral particle coordinates typically undergo the super-resolution analysis because the CCD camera records the lateral photon distribution in the 2-D pixel array. We showed here how to record the axial photon distribution using the translating CCD detector then determined the peak position of a point object with <10 nm precision (Figure 3). By this method we determined M_a_ linearly calibrating the image to the sample space axial dimension.

PSF shape also depends on light polarization linking it to the emitting probe dipole orientation. Probe orientation linkage to fluorescence spatial distribution has been demonstrated for back focal plane images [7], [8], defocused emission patterns [19], [20], wide field microscopy [16], and near-field scanning optical microscopy (NSOM) [4]. These methods extract more of the information content from the image compared to a more traditional orthogonal polarized intensity ratio, however, they require substantial intensity pattern analysis. The intensity pattern analysis conserves scarce photons from single molecule images that are lost in polarizing beam splitters separating the orthogonal polarization images. The latter is an important advantage in our application to GFP tagged myosin cross-bridges in muscle fibers.

After propagation through the microscope optics, we devolve the point image spatial pattern or PSF into the 6 basis patterns shown in Figure 4. In linear combination, they specify any single molecule emission pattern. Basis patterns are constant, for a given microscope and dipole distance from the glass/aqueous interface, and only their real coefficients change to fit an observed pattern image. Given the basis patterns, we invert an observed image to deduce their coefficients by using maximum likelihood fitting for Poisson distributed uncertainties. The coefficients for the basis patterns depend algebraically on the dipole orientation and establish the one-to-two correspondence between pattern and dipole moment orientations given by (θ_p,_φ_p_) and (π−θ_p,_π+φ_p_). We tested the method on simulated data then treated data from myosin cross-bridges in permeabilized muscle fibers to deduce single myosin lever arm exchanged RLC tagged PA-GFP orientation in rigor.

One example of simulated data is shown in Figure 6. The entire data set contains 70 patterns generated with normally distributed (θ_p,_φ_p_) angles for the dipole moment 50 nm from the glass/aqueous interface (in the aqueous phase) and with Poisson distributed noise from signal and two background sources. Figure 7A represents results for the dipole orientation distribution and Figure 7B demonstrates pattern sensitivity to the axial position of the probe relative to the nominal objective focus. Fitted patterns produce an accurate representation of the dipole orientation distributions and a somewhat less accurate representation of dipole spatial position. The situation improves for better S/N ratio data and when axial scanning data augments the data set.

Muscle fiber data is summarized in Figures 8, 9, 11. Figure 8 shows single molecule patterns, their fitted representation, and the respective residuals. The perpendicular polarized single molecule pattern sprawls beyond the fitted data suggesting the image is somewhat unfocused. This notion is supported by the skewed axial distribution observed for this set of probes shown in Figure 11B. The PA-GFP tagged muscle fiber object was brought into focus in the microscope with background fluorescence from the un-photoactivated probes, photoactivated with a bright pulse of polarized light, then refocused using the collection of single molecule images that appeared like a darkened but star filled sky. Specimen focus was judged by the overall impression of the image without selecting any single molecule images for special emphasis. Consequently, it is expected that some of the single molecules will be out of focus. Our data suggests sample defocus was prevalent in the perpendicular polarized photoactivated sample because most of these patterns were at the outer limit or beyond the interval in ε for which patterns were computed while for parallel polarized photoactivated molecules most are within limits. The perpendicular polarized photoactivated sample tends to produce photoactive molecules with dipoles perpendicular to the glass/aqueous interface. The perpendicular dipoles have a sprawling, donut shaped light intensity pattern that is inherently more difficult to focus.

**Figure 12:**
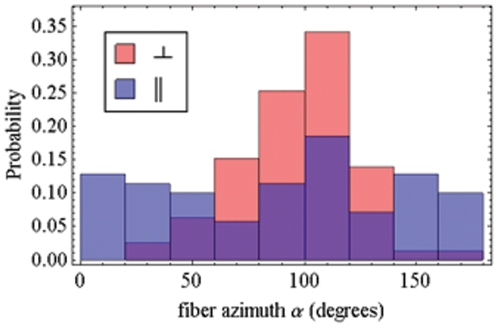
The probe azimuthal orientation distribution probability histograms. The orientation distribution in the azimuthal angle from probe coordinates, α, for perpendicular or parallel polarization (red or blue) photoactivated PA-GFP tagged muscle fibers in rigor. Angle α is a rotation about the fiber symmetry axis. The probe distribution suggests perpendicular, but not parallel, polarization photoactivation breaks the fiber symmetry.

Orthogonal polarized photoactivation laser pulses were applied to the PA-GFP tagged myosin cross-bridges in separate fiber samples to photoselect contrasted oriented sub-populations of probes that are intrinsically oriented by fiber structure. Evidence for both orientation selecting processes was seen in the data. Cross-bridge orientation distribution in the fiber azimuthal degree of freedom, α, is uniform while the parallel polarized photoactivated sample must photoselect a uniform distribution in α due to symmetry. In contrast, the perpendicular polarized photoactivated sample should have a non-uniform distribution in α despite the fiber myosin symmetry in this degree of freedom. These data are contrasted in Figure 9 but are more evident in the α-only projection of that data shown in Figure 12. The perpendicular polarized photoactivation (red) vs parallel polarized photoactivation (blue) samples show a strong contrast in the α-degree of freedom due to photoselection. Cross-bridge orientation distribution in the fiber polar angle, β, is strongly anisotropic. In perpendicular polarization photoactivated cross-bridges (Figure 9A in red) predominant angles are 45, 135 degrees while the photoactivating field polarization is 90 degrees clearly indicating the dominating presence of intrinsic fiber ordering. Parallel polarization photoactivated cross-bridges (Figure 9A in blue) showed a random distribution while an ordered distribution like that seen for the perpendicular photoactivation is expected. Modeling suggests this is the consequence of a statically disordered un-photoactivated absorption dipole, μ_a_[B], that disorients the photoactivated molecules. The PA-GFP chromophore is locally more flexible than GFP because it is highly accessible to quenchers [10], [21]. It undergoes a large conformation change to accomplish photoactivation also indicating a more flexible chromophore [22]. The disorder in μ_a_[B] is asymmetrical in the photoactivation of myosin cross-bridges (contrasting parallel and perpendicular photoactivation emission dipole orientation distributions) because the disordering retains the mostly perpendicular μ_a_[B] orientation distribution enabling perpendicular photoactivation and de-enabling parallel photoactivation. This effect is captured qualitatively by the model distribution shown in Figure 9B.

Probe axial position detection is an added benefit of intensity pattern fitting that has not been exploited experimentally. Simulations summarized in Figures 7B and 11A suggest probe axial position can be accurately assessed. A strategy utilizing axial image scanning decreased assessment errors for both axial and orientation distributions. Axial scanning is already a standard feature in confocal microscopy and is easily implemented with the piezo nanopositioner on the objective shown in Figure 1. Axial position detection of the PA-GFP tagged myosin cross-bridges in a muscle fiber was inconclusive (Figure 11B). Although there appears to be contrast between the patterns tested that is consistent with expectations for dipoles oriented perpendicular or parallel to the glass/aqueous interface (due to perpendicular or parallel photoactivation) more work needs to be done to assess whether it is a practical technique.

In conclusion, the lateral and axial PSF for a dipole emitter is sensitized to the emission polarization and dipole axial position providing links between the PSF pattern and dipole orientation and axial position. A general expression for the 3-dimensional PSF shows it is composed of 6 basis patterns that in linear combination can specify any single molecule emission pattern. Given the basis patterns, we invert an observed image to deduce their coefficients by using maximum likelihood fitting for Poisson distributed uncertainties. The coefficients for the basis patterns depend algebraically on the dipole orientation. We tested the method on simulated data then treated data from myosin cross-bridges in permeabilized muscle fibers to deduce single myosin lever arm exchanged RLC tagged PA-GFP orientation in rigor. Orthogonal polarized photoactivation laser pulses were applied to the PA-GFP tagged myosin cross-bridges in separate fiber fields to photoselect contrasted oriented sub-populations of probes that are intrinsically ordered by fiber structure. Evidence for both orientation selecting processes was detected in the data and quantified by dipole orientation distributions. Axial probe position dependence in the PSF was quantified in simulation but evidence of it from real data was inconclusive. A method utilizing axial scanning was indicated that boosts axial and orientational resolution in simulation.
